# Microglia Stimulation by Protein Extract of Injured Rat Spinal Cord. A Novel *In vitro* Model for Studying Activated Microglia

**DOI:** 10.3389/fnmol.2021.582497

**Published:** 2021-05-20

**Authors:** Joaquim Hernández, Isaac Francos-Quijorna, Elena Redondo-Castro, Rubén López-Vales, Xavier Navarro

**Affiliations:** Group of Neuroplasticity and Regeneration, Department of Cell Biology, Physiology and Immunology, Institute of Neurosciences, Centro de Investigación Biomédica en Red sobre Enfermedades Neurodegenerativas (CIBERNED), Universitat Autònoma de Barcelona, Red de Terapia Celular (TerCel), Bellaterra, Spain

**Keywords:** microglia, spinal cord injury, microglia culture, lysate, phagocytosis, mRNA expression, rat

## Abstract

Research on microglia has established the differentiation between the so-called M1 and M2 phenotypes. However, new frameworks have been proposed attempting to discern between meaningful microglia profiles. We have set up an *in vitro* microglial activation model by adding an injured spinal cord (SCI) lysate to microglial cultures, obtained from postnatal rats, in order to mimic the environment of the spinal cord after injury. We found that under the presence of the SCI lysate microglial cells changed their phenotype, developing less ramified but longer processes, and proliferated. The SCI lysate also led to upregulation of pro-inflammatory cytokines, such as IL-1β, IL-6, and TNF-α, downregulation of the anti-inflammatory cytokines IL-10 and IL-4, and a biphasic profile of iNOS. In addition, a latex beads phagocytosis assay revealed the SCI lysate stimulated the phagocytic capacity of microglia. Flow cytometry analysis indicated that microglial cells showed a pro-inflammatory profile in the presence of SCI lysate. Finally, characterization of the microglial activation in the spinal cord on day 7 after contusion injury, we showed that these cells have a pro-inflammatory phenotype. Overall, these results indicate that the use of SCI lysates could be a useful tool to skew microglia towards a closer phenotype to that observed after the spinal cord contusion injury than the use of LPS or IFNγ.

## Introduction

Spinal cord injury (SCI) leads to partial or complete loss of motor, sensory and autonomic functions below the injury level, due to damage to the local circuitry of the spinal cord and interruption of ascending and descending neural pathways (Ahuja et al., 2017). Traumatic SCI causes direct tissue damage that initiates a cascade of secondary events resulting in expanded tissue loss. There is a coordinated change in gene and protein expression profile associated with physiopathological events, including hemorrhage, excitotoxicity, oxidative stress, neuronal activity imbalance, and inflammation (Tator and Fehlings, 1991; Popovich, 2014; Siddiqui et al., 2015; Silver et al., 2015; Greenhalgh et al., 2020).

Microglia are one of the most important cells that participate in the inflammatory response that occurs after SCI. Although they are often considered to be macrophages of the CNS, recent studies documented microglia as a unique cell population, with distinct lineage and molecular signature than macrophages (Salter and Beggs, 2014). Microglia play many physiological functions in the CNS. For instance, they track tissue environment from insults or pathogens (Nimmerjahn, 2005), maintain tissue homeostasis and play a key role in the development of CNS (Prinz et al., 2019), control neuronal activity, synaptic maturation and plasticity, drive programmed cell death in CNS development, undergo central players in the innate immune response following injury (David and Kroner, 2011; Salter and Beggs, 2014).

Microglia rapidly change their phenotype in response to any disturbance of tissue homeostasis towards a commonly referred to as activated phenotype on the basis of changes in morphology or expression of cell surface antigens (Ransohoff and Perry, 2009; David and Kroner, 2011; Franco and Fernández-Suárez, 2015; Gensel and Zhang, 2015; Prinz et al., 2019). The specific activity of microglia after injury includes a wide range of functions, performed through specific roles of polarized microglia at different stages of the injury (Kigerl et al., 2009). It is usually considered as the concept of two different forms of microglia/macrophage polarization induced by either Th1 (IFNγ, LPS) or Th2 (IL-4 and IL-13) mediators. IFNγ/LPS-polarized microglia/macrophages are called M1 or “classically” activated microglia and macrophages and are characterized by expressing high levels of iNOS, TNF-α, or IL-1β, and the surface markers CD16/32. On the other hand, IL-4 or IL-13 promotes M2 or “alternatively activated” microglia and macrophage. M2 microglia/macrophages are characterized by expressing Arg1, CD206 and anti-inflammatory cytokines, such as IL-10 or TGF-β (Gordon and Taylor, 2005; David and Kroner, 2011; Francos-Quijorna et al., 2016). The current view of M1/M2 polarization is a simplified model that only represents two extremes of activation states (Ransohoff, 2016). However, after CNS injury, microglia are not stimulated by a single factor, such as IFNγ or IL-4, but by multiple mediators that lead microglia to adopt different activation states and functions to those observed in cell culture conditions. For this reason, there is a need to develop novel *in vitro* approaches that could drive microglia to a closer activation state to that observed *in vivo* after a CNS challenge. For this purpose, here we studied the effects of a spinal cord injury lysate on microglia activation as a more physiopathological approach than classical addition of IFNγ/LPS or IL-4. By studying microglia polarization *in vitro* under the effect of an SCI lysate, we aim to gain knowledge about microglia phenotype changes and to have a screening assay for comparison with the *in vivo* microglia reactivity after SCI.

## Materials and Methods

### Spinal Cord Injury

Adult female Sprague–Dawley rats (9 weeks old; 250–300 g) were used. The animals were housed with free access to food and water at a room temperature (RT) of 22 ± 2°C under a 12:12 light-dark cycle. The experimental procedures were approved by the ethical committee of the Universitat Autònoma de Barcelona in accordance with the European Directive 86/609/EEC. Under anesthesia with ketamine (90 mg/kg; Imalgene^R^ 1000; Boehringer Ingelheim, Germany) and xylazine (10 mg/kg; Rompun^R^; Bayer, Germany) and aseptic conditions, a longitudinal dorsal incision was made to expose the T6-T10 spinous processes. A laminectomy of T8-T9 vertebra was made and a cord contusion of 100 Kdynes was induced using an Infinite Horizon Impactor device (Precision System and Instrumentation, Fairfax Station, VA, USA). The wound was sutured by planes and the animals allowed to recover in a warm environment. An intraperitoneal (i.p.) bolus of saline solution (B.Braun Vetcare, UK) was administered immediately after surgery. To prevent infection, amoxicillin (500 mg/L; Normon, Spain) was given in the drinking water for 1 week. Postoperative analgesia was provided with buprenorphine (0.05 mg/kg; B.Braun Vetcare, UK) for 48 h. Bladders were expressed twice a day until reflex voiding was re-established.

Extracts were obtained from the intact spinal cord or from the injured spinal cord harvested at 7 days after the contusion. Fresh tissue was removed from the lesion site (around 1 cm), and an ultrasonic probe applied to disaggregate it in HBSS buffer (Gibco, USA). The extract was spun down (10,000 g) and filtered. Quantification of the protein content from the supernatant was made by the BCA method (Thermo Fisher Scientific, Germany), and a range of 15–20 μg protein/μl was obtained by colorimetry (Bio-Tek Instruments Inc., Germany). Extracts were water-soluble and were solubilized in HBSS buffer.

### Microglial Cultures

Glial cell cultures were prepared from 1-day-old Sprague–Dawley rats, as previously described (Redondo-Castro et al., 2013). Briefly, following the removal of meninges, brain tissue was minced and incubated for 10 min at 37°C in Ca^2+^-free Krebs-Ringer buffer containing 0.0025% trypsin (Sigma–Aldrich, St. Louis, MO, USA). Enzyme dissociated cells were mechanically triturated through a glass pipette and filtered through a 40-μm nylon mesh (Corning, USA) in the presence of 0.52 mg/ml soybean trypsin inhibitor (Gibco, USA) and 170 IU/ml DNAse (Roche, Switzerland). After centrifugation (500 g), the cells were stained with Trypan Blue exclusion dye (Sigma–Aldrich, St. Louis, MO, USA), counted in a Neubauer chamber, and then resuspended in 90% Dulbecco’s modified Eagle medium (DMEM; Gibco, USA), 10% foetal bovine serum (FBS; Sigma–Aldrich, St. Louis, MO, USA), 20 U/ml penicillin and 20 mg/ml streptomycin (Sigma–Aldrich, St. Louis, MO, USA) at 3 × 10^5^ cells/ml.

Cells were plated and incubated at 37°C in a humidified atmosphere of 5% CO_2_ and 95% air (Heracell™ 150i CO_2_ incubator; Thermo Fisher Scientific); medium was replaced every 3 days. After 14–15 days* in vitro*, the culture became confluent and microglial cells were obtained by shaking the flasks for 2 h at 200 rpm. Floating cells were spun down to obtain the dislodged cells and cultured at 150,000 cells/ml. This enriched fraction (around 95–97%) of microglia was seeded on poly-D-lysine-treated well or glass cover-slips in 24-well plates (PDL from Sigma–Aldrich, St. Louis, MO, USA), and maintained in DMEM medium supplemented with 10% FBS, 20 U/ml penicillin and 20 μg/ml streptomycin. After 3 days, microglial cells were ready to be treated.

Agents used for microglia activation were lipopolysaccharide at 10 ng/ml (LPS; *Escherichia coli* 0127:B Sigma–Aldrich, St. Louis, MO, USA), IL-4 at 10 ng/ml (Affymetrix eBioscience, USA), IFNγ at 10 ng/ml (Affymetrix eBioscience, USA) or an extract lysate obtained from the intact and 7 days injured spinal cords (SC lysate and SCI lysate). Different concentrations of the lysate were tested depending on the experiment (10, 25, 50 or 100 μg/ml).

### Immunocytochemistry

After treatments, coverslips were fixed in 4% paraformaldehyde for 20 min at RT and washed in PBS. Blockade of nonspecific binding was performed with 5% foetal calf serum (FCS) in 0.1% Triton X-100/PBS. Then, coverslips were incubated in the same solution at 4°C overnight with primary antibodies: anti-rabbit iba1 (1:500; Wako, Japan) or anti-goat iba1 (1:200; Abcam, Cambridge, MA, USA), used to detect macrophage/microglial cells; anti-mouse GFAP (1:200; Sigma–Aldrich, St. Louis, MO, USA), used to detect astrocytes; anti-rabbit Tubulin β-3 (1:200; Biolegend, USA), used to detect neurons; anti-mouse O4 (1:200; Sigma–Aldrich, St. Louis, MO, USA), used to detect oligodendrocytes; or anti-rabbit Ki67 (1:200; Abcam, Cambridge, MA, USA), used to detect proliferation. After several washes in 0.1% Triton X-100/PBS, samples were incubated for 2 h at RT with donkey anti-rabbit Alexa Fluor 488-conjugated antibody (1:200; Invitrogen, USA), donkey anti-mouse Alexa Fluor 488-conjugated antibody (1:200; Invitrogen, USA), donkey anti-goat Alexa 488-conjugated antibody (1:200; Invitrogen, USA), donkey anti-rabbit Cy3-conjugated antibody (1:200; Jackson IR, USA) or donkey anti-mouse Cy3-conjugated antibody (1:200; Jackson IR, USA), in 0.1% Triton X-100/PBS with 1.5% FCS at RT. In the last wash, cells were incubated with DAPI (5 μg/ml; Sigma–Aldrich, St. Louis, MO, USA) to perform nuclear staining.

### Morphological Analysis of Microglia

Microglia morphology was quantified in fluorescent images from Iba1-immunostained cultures by using ImageJ software (NIH, USA) and skeleton analysis plugin [Analyze Skeleton (2D/3D) from http://imageJ.net/AnalyzeSkeleton]. Analysis was performed according to a previously described procedure (Young and Morrison, 2018; Blasco et al., 2020). For this purpose, digital photomicrographs were transformed to 8-bit grayscale and then binarised to obtain a black and white image by means of a formerly established threshold. Every image was manually edited to obtain an image with a continuous set of pixels and gaps between processes belonging to neighboring cells. The image was then saved, and the plugin Skeleton (2D/3D) was run. This step results in a tagged skeleton image from which the number of endpoints and branch length can be summarized from the resulting output files. Endpoints and process length data are then used to estimate the range of microglia extensions in the photomicrograph. Four images per condition, from three biological replicates, were used to perform these analyses (ranging between 600 and 800 total cells per replicate).

### Proliferation Assay

At 24 h upon microglia stimulation, cells were fixed with 4% paraformaldehyde (PFA) for 15 min and immunohistochemistry against Iba1 (1:500; Wako, Japan) and Ki67 (1:200; Abcam, Cambridge, MA, USA) was performed as indicated above. Several images were randomly taken and total cells and labeled Ki67 cells were counted. Microphotographs were analyzed using ImageJ software (NIH, USA); Ki67 was represented as percentage vs. total cells. Three biological replicates per condition were used for these analyses.

### Survival Assay

Propidium iodide (PI) is a standard red-fluorescent probe enabling distinguishing viable cells from the dead ones. Cells were trypsinized and resuspended in 100 μl staining buffer containing PI (50 μg/ml; Sigma–Aldrich, St. Louis, MO, USA), and incubated for 15 min at RT in the dark (Calderwood and Prince, 2018). Once setting conditions were defined, samples were analyzed by flow cytometry (BD Bioscience, USA). There 10,000 events from each sample were analyzed. Dead cells were defined as PI+ cells. Percentages of PI+ cells were calculated from three biological replicates.

### Metabolic Activity

MTT, 3-(4,5-dimethylthiazol-2-yl)-2,5-diphenyltetrazolium bromide (Sigma–Aldrich, St. Louis, MO, USA), was used to evaluate mitochondrial activity as an indirect measure of cell metabolic activity, as previously described (Redondo-Castro et al., 2013; Alé et al., 2015). Microglial cells were incubated with LPS, extract lysate or extract lysate with ibuprofen (Sigma–Aldrich, St. Louis, MO, USA). After 24 h incubation, 0.15 mg/ml of MTT was added and the cells were incubated for 3 h at 37°C. The formazan crystals were dissolved in 200 μl of dimethyl sulfoxide (DMSO; Sigma–Aldrich, St. Louis, MO, USA) and 150 μl were passed to 96-well plates. The optimal density was determined with a microculture plate reader (Bio-Tek Instruments Inc., Germany) at 570 and 620 nm (to counteract the noise of the plastic) and analyzed with KCjunior™ software (Bio-Tek Instruments Inc., Germany). Three biological replicates were done.

### RNA Isolation, Reverse Transcription, and Real-Time PCR

Cells were collected in RTL buffer reagent (Qiagen, Germany) and RNA extracted using the RNeasy Mini kit (Qiagen, Germany), according to the protocol of the manufacturer. RNA was treated with DNase I (Qiagen, Germany) to eliminate genomic DNA contamination. One microgram of RNA obtained from microglia was primed with random hexamers (Promega, USA) and reverse transcribed using the Omniscript RT kit (Qiagen, Germany). RNase inhibitor (Roche, Switzerland) was added (1 U/μl final concentration) to avoid RNA degradation. Primer sequences for real-time PCR are specified in Table 1. Glyceraldehyde-3-phosphate dehydrogenase (GAPDH) was used as a housekeeping gene. Gene-specific mRNA analysis was performed by SYBR-green real-time PCR using the MyiQ5 real-time PCR detection system (Bio-Rad Laboratories, Spain). Previously, we had determined the optimal concentration of the cDNA to be used as a template for each gene analysis to obtain reliable CT (threshold cycle) values for quantification (between 800 ng and 1 μg/μl). The thermal cycling conditions comprised 3 min polymerase activation at 95°C, 40 cycles of 10 s at 95°C for denaturation and 30 s at 60°C for annealing and extension (62°C in the case of IL-6), followed by a DNA melting curve for determination of amplicon specificity. CT values were obtained and analyzed with BioRad Software. Fold change in gene expression was estimated using the CT comparative method (2^−ΔΔCT^; Livak and Schmittgen, 2001) normalizing to GADPH CT values and relative to control samples. Three samples were used per condition from three to five biological replicates.

**Table 1:** Primer sequences for real-time PCR analysis.

Gene (*Rattus norgevicus*)	Accession number	Primer sequence 5′-3′	Size	Product size (bp)
GAPDH	XM_573304.3	Forward: AATTCAACGGCACAGTCAAGGC	22	116
		Reverse: TACTCAGCACCGGCCTCACC	20	
Interleukin-1β	NM_031512.2	Forward: TCCCAAACAATACCCAAAGAAG	22	162
		Reverse: CCGACCATTGCTGTTTCC	18	
Interleukin-6	NM_012589.1	Forward: ATCTGCCCTTCAGGAACAGCTATG	24	110
		Reverse: ACTTGTGAAGTAGGGAAGGCAGTG	24	
Interleukin-10	X60675.1	Forward: TCCTTTCACTTGCCCTCATC	20	159
		Reverse: CGAGACTGGAAGTGGTTGC	19	
TGF-β1	NM_021578.2	Forward: AGGACCTGGGTTGGAAGTG	19	127
		Reverse: GTGTTGGTTGTAGAGGGCAAG	21	
TNF-α	NM_012675.3	Forward: GCGTGTTCATCCGTTCTC	18	190
		Reverse: CAGCGTCTCGTGTGTTTC	18	
Arginase 1	BC091158.1	Forward: TGGAACGAAACGGGAAGG	18	118
		Reverse: CTGGTTCTGTTCGGTTTGC	19	
iNOS	NM_012611.3	Forward: GAGTGAGGAGCAGGTTGAG	19	158
		Reverse: TGCTGTAACTCTTCTGGGTG	20	
IL-4	NM_201270.1	Forward: AACAAGGAACACCACGGAG	19	200
		Reverse: TTCAGTGTTGTGAGCGTGG	19	

### Phagocytosis Assay

One day after the treatments, cells were incubated with latex microbeads (1 μm, 0.0025%, Sigma–Aldrich, St. Louis, MO, USA) for 30 or 120 min at 37°C. After this, the medium was removed, and the cells rinsed twice with glucose-phosphate-buffered saline (PBS) to wash out the free beads. Then cells were fixed with 4% paraformaldehyde (PFA) for 15 min. After rinsing, labeling against Iba1 (1:500; Wako, Japan) was used. Several images were randomly taken and the number of cells with beads inside as well as the number of beads in each cell were counted. A minimum of 300 cells were measured for each condition, from three independent biological replicates, using ImageJ software (NIH, USA); the number of beads per cell and percentage of cells with beads were calculated (based on Silva et al., 2011).

### Fluorescent Activated Cell Sorting (FACS) Analysis

The cultured cell suspension was centrifuged twice at 300 g for 10 min at 4°C with DMEM containing 10% Foetal Bovine Serum (FBS). Primary antibody labeling was performed for 1 h at 4°C. Cells were labeled with mouse anti-rat conjugated antibody CD45-FITC (1:250; BD Bioscience, USA). For intracellular analysis of iNOS and ArgI, cells were fixed, washed, permeabilized and incubated with rabbit antibodies against iNOS (1:250; Abcam, Cambridge, MA, USA) and goat antibodies against ArgI (1:200 Santa Cruz, CA, USA). After 30 min of incubation, cells were washed and stained with PE (1:500; Affymetrix eBioscience, USA) or Alexa647 (1:500; Abcam, Cambridge, MA, USA) conjugated donkey secondary antibodies against goat or rabbit respectively for 30 min. Finally, the samples were washed and fixed in 1% PFA.

Immune cells from the injured spinal cord were analyzed by flow cytometry. Rats were terminally anaesthetized with an overdose of sodium pentobarbital and intracardially perfused with PBS. The spinal cord thoracic segment was harvested, mechanically triturated and then passed through a cell strainer of 70 μm (BD Bioscience, USA), with DMEM containing 10% of FBS. The cell suspension was centrifuged at 300 g for 10 min at 4°C. Samples were split into several tubes and immunostained. Primary antibody labeling was performed for 1 h at 4°C, using DMEM+10% FBS as a buffer. Cells were labeled with the following mouse anti-rat conjugated antibodies: CD45-PE-Cy7 (1:250; BD Bioscience, USA) and CD11b-FITC (1:250; BD Bioscience, USA). For intracellular staining, cells were fixed with 4% PFA and permeabilized with Permeabilization Wash Buffer (Invitrogen, USA), followed by staining with unconjugated rabbit antibodies against iNOS (1:200; Abcam, Cambridge, MA, USA) and goat antibodies against Arg1 (1:150; Santa Cruz, CA, USA), and rabbit (1:1,000 Wako, USA) or goat (1:500 Abcam, Cambridge, MA, USA) antibodies for Iba1 for 45 min. After that, we stained with PE (1:500; Affymetrix eBioscience, San Diego, USA) or Alexa 647 (1:500; Abcam, Cambridge, MA, USA) conjugated donkey secondary antibodies against goat or rabbit respectively for 30 min. The following combination of markers was used to identify microglia (CD45low, CD11b+), peripheral myeloid cells (CD45high, CD11b+), as previously described (Francos-Quijorna et al., 2016). Peripheral myeloid cells were further gated for Iba-1 to distinguish macrophages (CD45high, CD11b+, Iba-1+) from neutrophils since Iba-1 is not expressed in granulocytes. Cells were analyzed on a fluorescent activated cell sorting (FACS) Canto flow cytometer (BD Bioscience, USA) and the results analyzed using FlowJo^®^ software version 10.0.7 (BD Bioscience, USA).

### Statistical Analysis

Data are shown as the mean ± SEM. Statistical comparisons between groups were made using two-way ANOVA with Bonferroni *post hoc* test in mRNA studies. One-way ANOVA with Tukey *post hoc* test was used for the assessment of cell counts, MTT assay, proliferation assay, survival assay, phagocytosis studies and microglial phenotype *in vitro*. Finally, *t*-test analyses were used in microglia morphology analysis and phenotype studies *in vivo* (IBM SPSS Statistics software, USA). Differences between groups were considered statistically significant at *p* < 0.05.

## Results

### Purity of Cultured Cells

**Figure 1:**
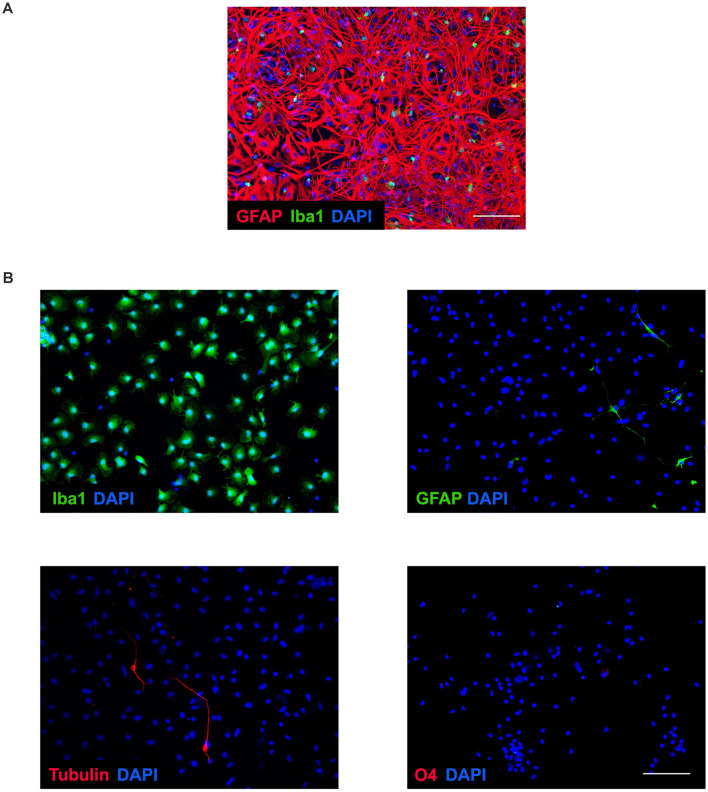
Microglia purity. **(A)** Representative image of confluent and mixed culture after 14 days* in vitro*. Immunochemical image outlining labeling of GFAP (red), Iba1 (green), and DAPI (blue). Microglial cells were obtained by shaking of mixed culture. Scale bar: 100 μm. **(B)** Representative images of enriched microglial culture showing labeling of Iba1 (green), GFAP (green), tubulin β-3 (red), O4 (red), and DAPI (blue). Scale bar: 100 μm.

Mixed cell culture exhibits a network of cells, mainly astrocytes, with microglia around not firmly attached to the plate at 14 days* in vitro* (Figure 1A). However, once microglia were purified by shaking the mixed culture, this was highly enriched in microglial cells and we only observed occasional negligible cell contamination, mainly by astrocytes and neurons (Figure 1B).

### Effect of SCI Lysate on Microglial Morphology, Metabolic Activity, Proliferation and Survival

**Figure 2:**
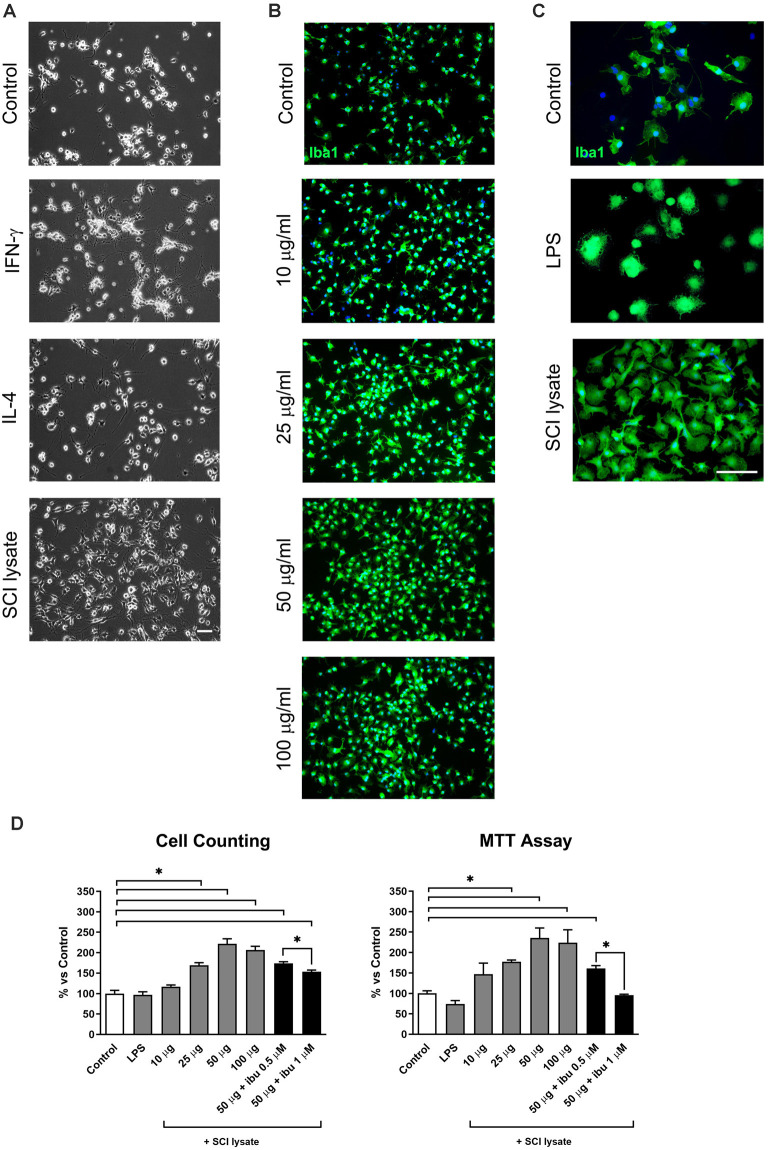
Effects of lipopolysaccharide (LPS) and spinal cord injury (SCI) lysates in microglial cell count and metabolic activity. **(A)** Representative images of microglial cells activated by IFN-γ at 10 ng/ml, IL-4 at 10 ng/ml and SCI lysate at 50 μg/ml, 24 h after treatment. Scale bar: 50 μm. **(B)** Immunochemical images depicting labeling of Iba1 (green) and DAPI (blue) with increasing concentration of SCI lysate in the medium at 10, 25 and 50 μg/ml. Scale bar: 100 μm.** (C)** Higher magnification images of control microglia, and microglia activated by LPS or SCI lysate (50 μg/ml), labeled with anti-Iba1 (green) and DAPI (blue). Scale bar: 100 μm. **(D)** Graph showing microglia cell counts and MTT activity at 24 h upon being exposed to LPS (10 ng/ml), different concentrations of SCI lysate (10, 25, 50 and 100 μg/ml), and a combination of SCI lysate (50 μg/ml) with ibuprofen at 0.5 and 1 μM. Values are expressed as mean and SEM (*P < 0.05). *n* = 3 biological replicates per condition.

Resting microglial cells in culture displayed a small round morphology with some short processes (Figures 2A–C). Activation with IFNγ induced many ramified extensions compared with the processes appearing with IL-4 activation (Figure 2A). The addition of lysates from the injured spinal cord produced qualitatively greater cell counts and morphological changes (Figure 2).

We therefore, assess the effects of the SCI lysates in cell counts, metabolic activity, and morphological changes. These experiments revealed that microglial cell counts increased after the stimulation with the SCI lysates in a concentration-dependent manner (Figures 2B–D), reaching a plateau at 50μg/ml concentration (Figures 2B,D). The effect of the SCI lysate on microglia metabolic activity was examined by MTT. When analyzing MTT activity in microglial we found that the addition of SCI lysate produced a significant increase in MTT activity (Figure 2D). Interestingly, ibuprofen, which is an anti-inflammatory drug (Redondo-Castro and Navarro, 2014), reduced the effects of the SCI lysates on microglial cell counts and MTT activity (Figure 2D). No changes in cell counts and MTT activity were observed in microglia upon LPS stimulation (Figure 2D).

Since microglia counts and MTT activity reached maximum levels at 50 μg/ml concentration of the SCI lysate, this concentration was used for the following studies.

**Figure 3:**
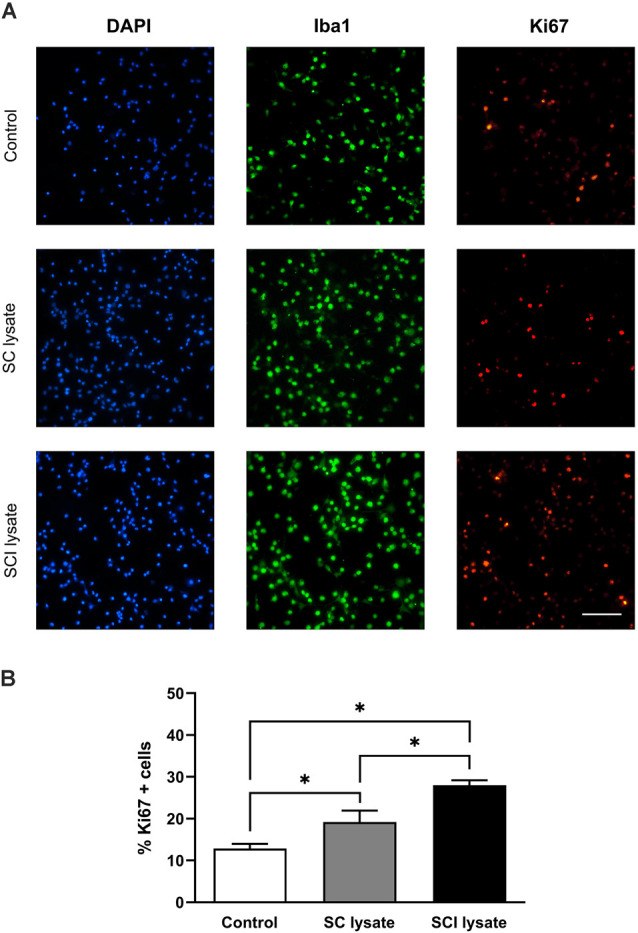
Effects of injured spinal cord (SCI) lysates in microglial proliferation. **(A)** Representatives immunocytochemical images of microglial cells activated by SC and SCI lysate at 50 μg/ml, 24 h after treatment. Images depicting labeling of Iba1 (green), DAPI (blue), and Ki67 (red). Scale bar: 100 μm. **(B)** Results from counting Ki67 + cells after treatments. Values are expressed as mean and SEM (*P < 0.05). *n* = 3 biological replicates per condition.

**Figure 4:**
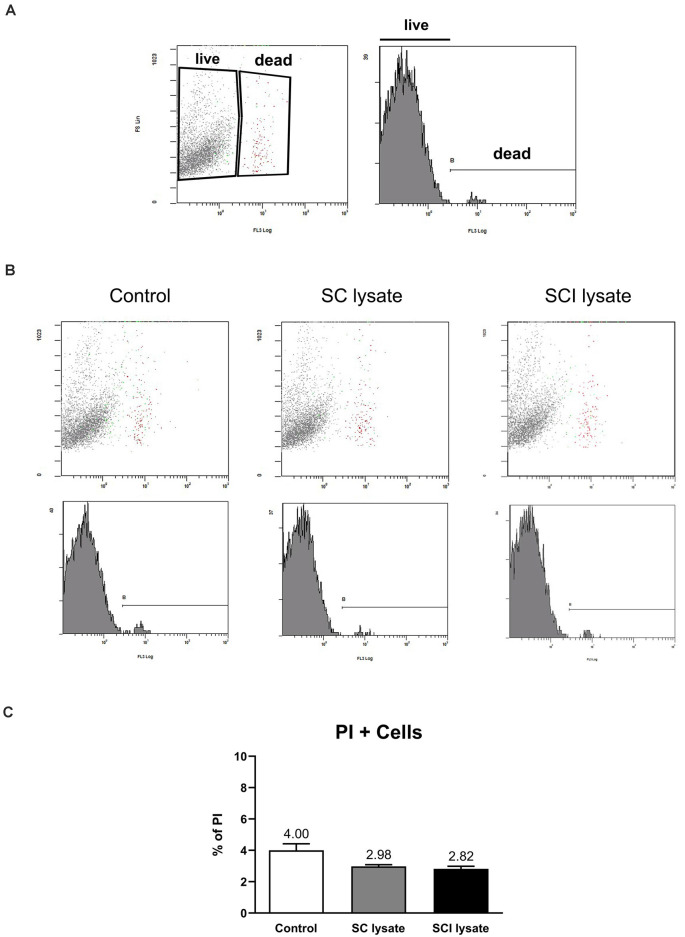
Effects of SCI lysates in microglial cell death. **(A)** Representative setting from propidium iodide (PI) in fluorescent activated cell sorting (FACS) density plot and histogram, showing how live and dead microglia were gated. **(B)** Representative plots from control and microglial cells activated by SC and SCI lysate at 50 μg/ml, 24 h after treatment. **(C)** Graphs showing the quantification of **(B)** expressed as PI+ cells (dead cells in each condition). Values are expressed as mean and SEM. *n* = 3 biological replicates per condition.

We assessed whether the effect of the SCI lysates on microglia counts was due to the ability of the lysate to induce cell proliferation (Figures 3A,B). Immunostaining against Ki67 revealed that the SCI lysate triggered a significant proliferation of microglia (Figure 3B). However, the lysate did not lead to microglial cell death (Figures 4A–C).

**Figure 5:**
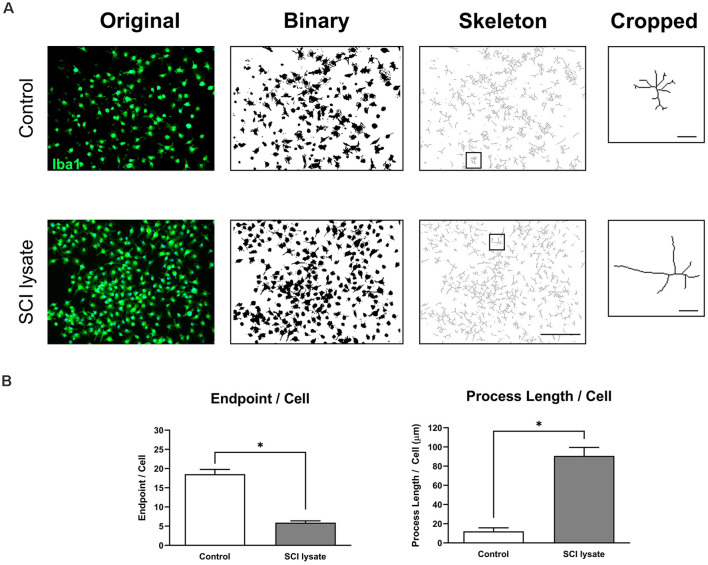
Effects of SCI lysates in microglia morphology. **(A)** Example of original photomicrograph from control and cells treated with SCI lysate (50 μg/ml), and conversion to binary and skeletonized image. Cropped cell corresponds to the box in the skeleton image. Scale bar: 100 μm. Scale bar: 20 μm in cropped images. **(B)** Summary data of microglia endpoints/cell and process length/cell in control and after SCI lysate treatment. Values are expressed as mean and SEM (*P < 0.05). *n* = 3 biological replicates per condition.

We then assessed the morphological changes induced by the SCI lysate in microglia. These analyses revealed that microglia became less ramified by the SCI lysate as revealed by the number of endpoints per cell (Figure 5A). However, the length of these processes was markedly increased by the lysate (Figure 5B).

### mRNA Expression in Microglial Cultures Treated With SC and SCI Lysates

In order to study the influence of SC and SCI lysate (50 μg/ml) on the expression of inflammatory mediators in microglial cells, we analyzed the transcript levels of IL-1β, IL-4, IL-6, IL-10, TNF-α, TGF-β1, and iNOS and Arginase 1 at different time points (0, 2, 4, 8, 12 and 24 h) as shown in Figure 4.

**Figure 6:**
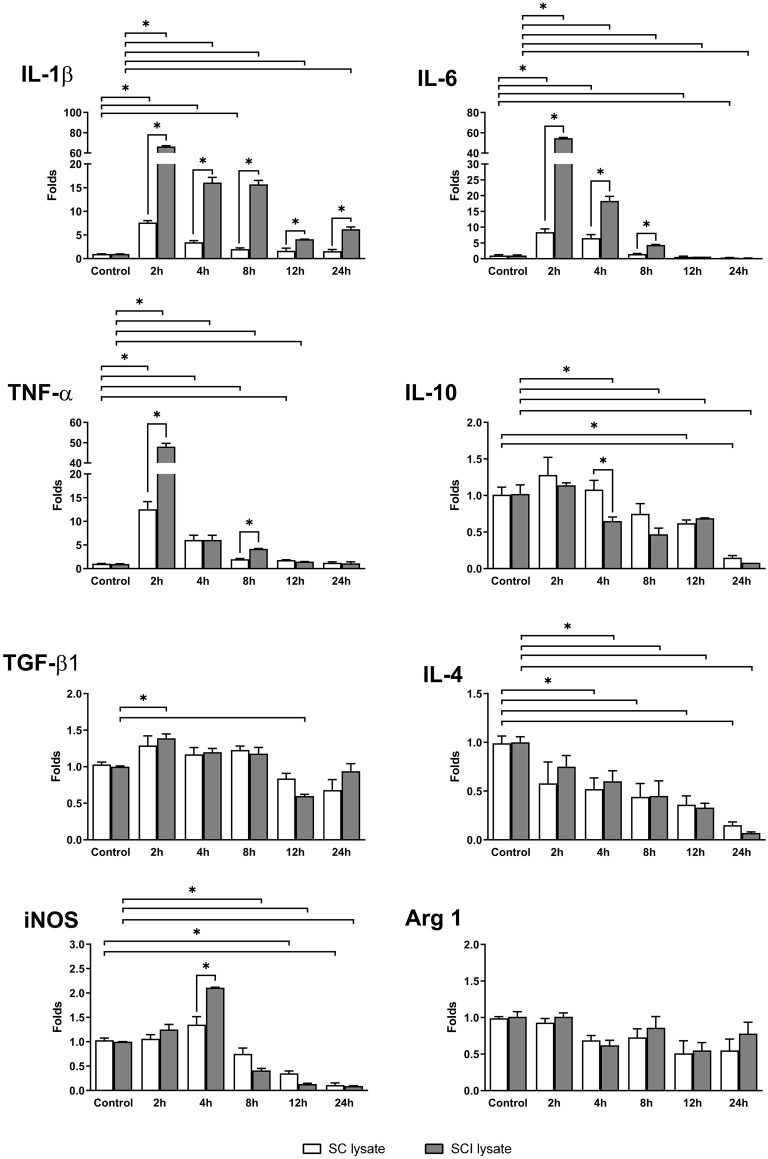
mRNA expression from microglia cultures. Real-time PCR analyses of different mRNAs expressed in microglia cultures, with the addition of SC lysate and SCI lysate (at 50 μg/ml). Samples were taken in a time-course culture at 0, 2, 4, 8, 12 and 24 h after treatment. Values are expressed as mean and SEM (*P < 0.05). *n* = 3–5 biological replicates per condition.

The transcripts of the pro-inflammatory cytokines IL-1β, IL-6, and TNF-α reached peak expression at 2 h upon stimulation and then decreased with time. The addition of the non-injured SC lysate produced a significant increase of the same pro-inflammatory cytokines, but to a much lower extent as compared to SCI lysate (Figure 6). Oppositely, the anti-inflammatory cytokines IL-10 and IL-4 were downregulated significantly by the addition of SCI lysate, whereas TGF-β1 showed a slight, but significant, upregulation at 2 h, and then a downregulation at 12 h (Figure 6). The reduced expression of IL-4 and IL-10 in microglial cells was also observed by the addition of the SC lysate, however, this was observed at later time points as compared to the SCI lysate.

Interestingly, inducible nitric oxide synthase (iNOS), a marker of pro-inflammatory microglia, showed a biphasic expression after the addition of the SCI, showing a significant increase in mRNA expression at 4 h and a later significant decrease from 8 h to 24 h. This early up-regulation in iNOS expression mediated by the SCI lysate was not observed by the addition of the SC lysate. Finally, the transcripts of Arginase 1 in microglia did not change upon SCI or SC lysate stimulation. These data provide clear evidence indicating that the SCI lysate drives microglia to adopt a pro-inflammatory phenotype.

### Microglial Phagocytosis

**Figure 7:**
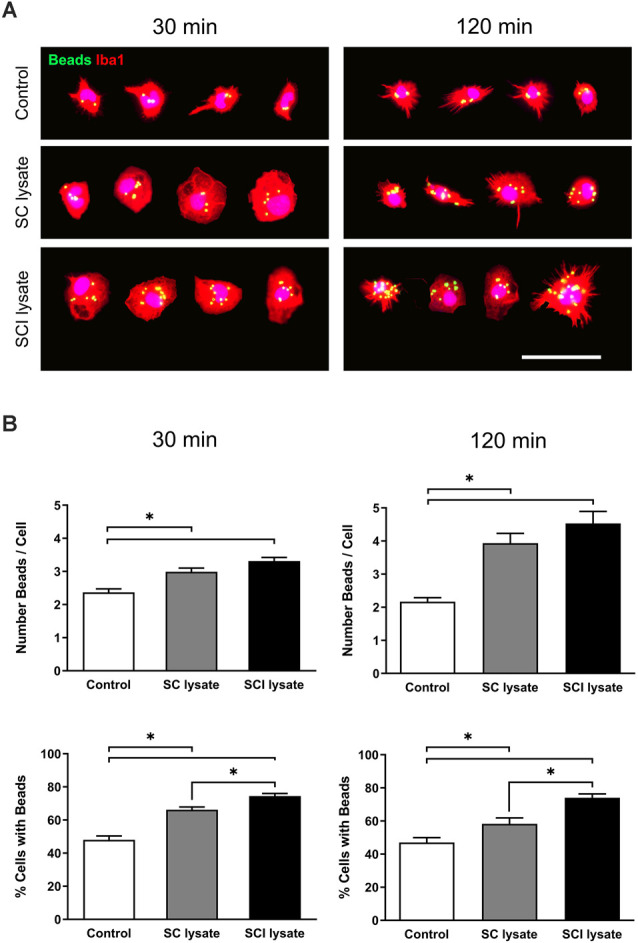
Effects of SCI lysates in microglia phagocytosis. **(A)** Representative images of the phagocytic assay (at 30 and 120 min), showing microglial cells labeled against Iba1, and green-labeled beads inside. Cell images are from control, SC lysate and SCI lysate treatment (at 50 μg/ml). Scale bar: 50 μm. **(B)** Results from phagocytic assay expressed as the mean of beads engulfed per cell in each condition, and percentage of cells with ingested beads, at 30 and 120 min after treatments. Values are expressed as mean and SEM (*P < 0.05). *n* = 3 biological replicates per condition.

A latex bead phagocytosis assay was performed in microglial cultures in the presence of SC and SCI lysates. Between 300 and 900 cells were analyzed from three independent experiments to avoid variability in the number of engulfed beads. Both lysates produced an increase in the number of beads detected inside the microglial cells. Representative images are shown in Figure 7A. Whilst in the control conditions, we observed that microglia engulfed approximately two beads per cell, either at 30 or 120 min after adding beads in the culture medium, microglia phagocytosed an average of four and five beads per cells after the addition of the SC and SCI lysate, respectively (Figure 7B). No significant changes were found in the number of engulfed beads per microglia after the addition of SC or SCI lysates. However, the SCI lysate significantly increased the percentage of microglia that phagocytosed beads as compared to the SC lysate (Figure 7B).

### Activated Microglial Pattern *In vitro*

**Figure 8:**
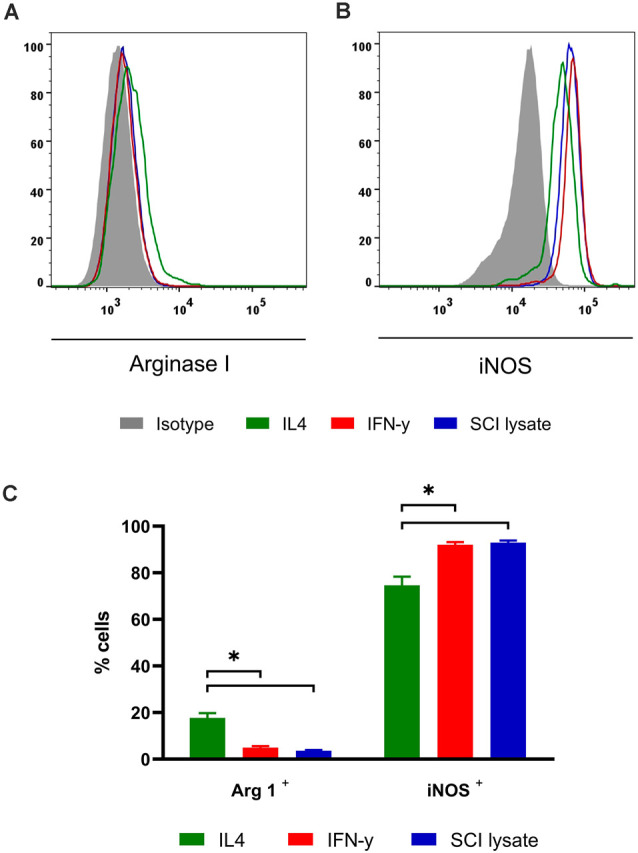
Effects of SCI lysates in microglia phenotype *in vitro*. **(A,B)** Representative FACS plots Arg1 and iNOS levels in cultured microglial cells. **(C)** Graph showing the quantification of cultured microglial cells expressing ArgI and iNOS after IFN-γ at 10 ng/ml, IL-4 at 10 ng/ml and SCI lysate at 50 μg/ml activation. Values are expressed as mean ± SEM (*n* = 4 biological replicates per condition; *P < 0.05).

We analyzed the effects of microglial activation by IFNγ, IL-4 or SCI lysate *in vitro* using flow cytometry. IFNγ was used as a positive control for the pro-inflammatory phenotype, while IL-4 was used as positive control for the anti-inflammatory profile. At 24 h after the treatment, the expression of Arg1 was barely detectable in microglial cells stimulated with IFNγ or the SCI lysate, whereas ~20% of the cells expressed Arg1 upon IL-4 treatment (Figures 8A–C). In contrast, ~90% of microglia expressed iNOS upon IFNγ or SCI lysate stimulation, whereas this enzyme was observed in ~75% of microglia after IL-4 (Figures 8B,C). Therefore these data indicate that phenotypically, microglial cells exposed to the SCI lysate were more similar to microglia stimulated with IFN-γ, and thus, further support the microglia adopt a pro-inflammatory phenotype after the addition of the SCI lysate.

### Microglial Phenotype After Spinal Cord Injury Analyzed by Flow Cytometry

**Figure 9:**
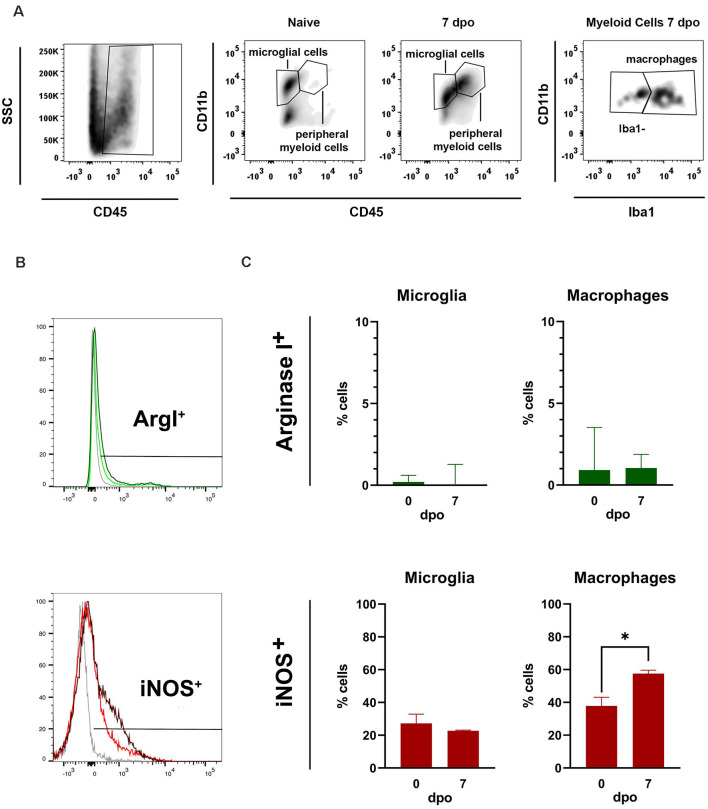
Flow cytometer analysis of microglia* in vivo*. **(A)** Representative fluorescent activated cell sorting (FACS) density plots of naïve and injured spinal cord showing how microglia and macrophages were gated (>10,000 cells cd45+ cd11b+). **(B)** FACS histogram plots showing iNOS and Arg1 expression in microglia and macrophages. **(C)** Graphs showing the quantification of **(B)** microglia and **(C)** macrophages expressing Arg1 and iNOS markers 7 days post operation (dpo). Note that the percentage of microglia and macrophages expressing iNOS is markedly higher than those expressing Arg1. Values are expressed as mean ± SEM (*n* = 3 per group; *P < 0.05).

By using FACS, we assessed the changes in expression of iNOS and Arg1 markers in microglia and macrophages 7 days after spinal cord contusion injury in the rat. We found that microglia and macrophages expressed mainly the pro-inflammatory marker iNOS after SCI, whereas the expression of anti-inflammatory marker (Arg1) was restricted to a very small population of microglia and macrophages (Figure 9). The expression of iNOS was detected in a low proportion of microglial cells in the uninjured and injured spinal cords (~27%). However, the proportion of iNOS+ macrophages increased significantly at 7 days post-injury as compared to those found in physiological conditions at very low numbers. These results, together with previous reports in the literature, indicate that microglia and macrophage are skewed towards a more pro-inflammatory phenotype.

## Discussion

Spinal cord injury produces an inflammatory response that includes the rapid activation of microglia and their release of pro-inflammatory mediators (Alexander and Popovich, 2009; David et al., 2015). Events that occur after spinal cord injury are characterized by hemorrhage, apoptosis, inflammation, and changes in the blood-spinal cord barrier and the extracellular matrix. All these processes produce a harmful and inhibitory environment that impairs endogenous regeneration and remyelination (Siddiqui et al., 2015). In addition, the balance of inflammatory and intrinsic repair processes influences the resolution of the neuroinflammatory reaction (DiSabato et al., 2016). The immune cell subsets, as major players involved in the immune response, play a key role in the pathological events after SCI (Plemel et al., 2014; David et al., 2015).

The microenvironment of the injured spinal cord in the acute phase contains many mediators, such as cytokines, chemokines, reactive oxygen species and secondary messengers (DiSabato et al., 2016). The SCI lysate obtained 7 days after lesion contains a profile of cytokines, pro-inflammatory mediators and other factors that come from the extracellular matrix and cellular contents of the lesion site and surrounding. We used this cocktail to study the response of microglia in culture. Microglia responded rapidly to the addition of the SCI lysate, showing marked changes in morphology, activity, and proliferation. Changes in microglia under the presence of SCI lysate in the medium were compared with classical administration of LPS, or the addition of IL-4 and IFNγ. In summary, SCI lysate induces changes in cell morphology (less ramified but longer processes), increases proliferation but does not affect survival. In a previous study, we studied the phenotype of neural stem cells (NSCs) derived from human induced pluripotent stem cells (iPSCs) under the presence of an SCI lysate (Lopez-Serrano et al., 2016), showing increased proliferation of iPSC-derived NSCs without changes in their immature stage, due to unknown factors present in the SCI lysate. It is well known that ischemia and immune infiltration can lead to oxidative stress and free radical production in the lesion area (Donnelly and Popovich, 2008). Ionic dysregulation following SCI contributes to cell damage and loss (Vosler et al., 2009). Furthermore, increasing glutamate levels, upregulating production of cytokines, matrix metalloproteinases (MMPs) or superoxide dismutase result ultimately in excitotoxic cell death (Donnelly and Popovich, 2008; Siddiqui et al., 2015; DiSabato et al., 2016).

The addition of a SCI lysate was a strategy to mimic the* in vivo* environment under controlled culture conditions. While microglia have been classically classified as either resting or activated (Streit et al., 1988), recently, some reports have used transcriptome profiles to identify genes selectively expressed in microglia. This microglial signature revealed a unique combination of genes, distinct from other immune cells (Wes et al., 2016). Our results revealed the response of microglial cells to an SCI lysate *in vitro*, showing a specific mRNA expression pattern, with a rapid and significant increase of IL-1β, IL-6, and TNF-α expression, downregulation of IL-10 and IL-4, and no changes in Arginase 1. iNOS showed a biphasic response with an early upregulation and a late downregulation. This cytokine profile is dynamic in time, between 2 and 24 h, indicating a phenotypic adjustment to the environment. The whole expression profile clearly indicates a proinflammatory phenotype, in agreement with other studies (David and Kroner, 2011). Microglia exposed to a non-injured SC lysate showed also a specific mRNA pattern profile. In this case, the increase in pro-inflammatory cytokines was transient and restricted in time, due to the SC lysate content, without free radicals and oxidative stress compounds. The* in vitro* phagocytosis assay (Redondo-Castro et al., 2013) revealed in our study that the SCI lysate induced a phagocytic phenotype in the microglia, indicated by the high number of cells with engulfed beads, compared with control cells or microglia with the SC lysate.

We also analyzed the microglial profile *in vitro* by flow cytometry. iNOS and Arg1 intracellular markers were used to characterize the microglia phenotype after CD11b^+^ CD45^+^ gating. In the presence of SCI lysate microglial cells showed an increased iNOS expression, and a slight decrease in Arg I expression, results that were similar to those found with IFN-γ treatment and in agreement with previous studies in macrophages and microglia (Mantovani et al., 2004; Franco and Fernández-Suárez, 2015). Thus, the SCI lysate promotes the microglia to adopt a proinflammatory phenotype as similar to what takes place *in vivo* after injury or *in vitro* after IFN-γ exposure.

To further analyze the proportion of microglial cells expressing each phenotypic marker we used flow cytometry analysis. Flow cytometry has been used in different studies to quantify or characterize microglia from *in vivo* samples. In one of the first studies, two cell-isolation methods based on mechanical dissociation and Percoll gradient separation were performed, and thereafter CD11b, CD45, and αβ T-cell receptor antibodies were employed for analysis (Campanella et al., 2002). An SCI model was used to analyze the resolution of acute inflammation by Prüss et al (Prüss et al., 2011), defining the existence of a resolution plateau index and characterizing the non-resolving aspects of inflammation after CNS injury. Some methods have been published to isolate microglia from mouse brain in order to assay expression profile, based in separation of microglia with discontinuous Percoll gradient and flow cytometry isolation using CD45 (Cardona et al., 2006), a modified protocol using magnetic bead purification and CD11b and CD45 markers by FACS (Chiu et al., 2013), or Percoll gradient plus CD11b staining with magnetic beads (Nikodemova and Watters, 2012). More recently, a modified method was published using Percoll gradient followed by CD11b staining to obtain an enriched neural cell suspension, in order to assess the ratio of proinflammatory and antiinflammatory cells, a useful tool to study neuroinflammation in traumatic CNS injury (Bedi et al., 2013). The cell suspension was identified by flow cytometry with CD11b+ CD45^lo^ and categorized as proinflammatory or antiinflammatory macrophage/microglia based on CD206 and F_c_γRII/III expression.

Our studies on *in vivo* samples included CD11b and CD45 staining to gate properly the population of microglia (CD45^low^, CD11b^+^) or peripheral myeloid cells (CD45^high^, CD11b^+^), as in previous studies in mice (Francos-Quijorna et al., 2016). Macrophages were gated from the peripheral myeloid cells based on the expression of Iba1, since this marker is not expressed in neutrophils. Microglia and macrophages in mice express predominantly proinflammatory markers for the first 2 weeks after SCI, whereas the expression of M2 is scarce (Kigerl et al., 2009; Francos-Quijorna et al., 2016). Here, we observed that microglia/macrophages profile on day 7 after contusion injury in rats was characterized by an increased iNOS+ expression in macrophages, and minimal expression of Arg1+ macrophages and microglial cells, suggesting that these cells also adopt a proinflammatory phenotype similar to that observed after spinal cord injury in mice. In addition, this proinflammatory phenotype was also acquired *in vitro* when microglia were exposed to the SCI lysate.

Many endogenous factors appear involved in the phenotype switching of inflammatory cells after CNS injury, including cytokines, microRNA, or STAT molecules (Hu et al., 2014; Akhmetzyanova et al., 2019). Effective models to approach the SCI microenvironment will allow at investigating signaling pathways, and further identification of regulatory molecules and microglia profiles that could ultimately accelerate research towards clinical applicability. Here, we show that SCI lysate could be an effective strategy to mimic the SCI milieu conditions and a useful tool for studying microglia activation.

## Data Availability Statement

The raw data supporting the conclusions of this article will be made available by the authors, without undue reservation.

## Ethics Statement

The animal study was reviewed and approved by Ethical committee of the Universitat Autònoma de Barcelona in accordance with the European Directive 86/609/EEC.

## Author Contributions

JH, RL-V, and XN designed the study. JH, IF-Q, and ER-C performed the research, analyzed or interpreted results. JH, IF-Q, RL-V, and XN wrote the manuscript. All authors contributed to the article and approved the submitted version.

## Conflict of Interest

The authors declare that the research was conducted in the absence of any commercial or financial relationships that could be construed as a potential conflict of interest.
